# Editorial: Predictive mechanisms in action, perception, cognition, and clinical disorders

**DOI:** 10.3389/fnhum.2022.1005905

**Published:** 2022-08-31

**Authors:** Anila M. D'Mello, Patric Bach, Philip R. Corlett, Liron Rozenkrantz

**Affiliations:** ^1^McGovern Institute for Brain Research, Massachusetts Institute of Technology, Cambridge, MA, United States; ^2^School of Psychology, University of Aberdeen, Aberdeen, Scotland; ^3^Department of Psychiatry, Yale University, New Haven, CT, United States; ^4^Department of Psychology, Yale University, New Haven, CT, United States; ^5^Wu Tsai Institute, Yale University, New Haven, CT, United States; ^6^The Azrieli Faculty of Medicine, Bar-Ilan University, Safed, Israel

**Keywords:** action, perception, cognition, disorders, predictive frameworks, prediction

The world is a noisy place, and people must decide which inputs are meaningful, which are uninformative, and how to use incoming information to optimize their behavior in different contexts. It has been argued that rather than passively processing incoming information, we use past experience to form predictions about what is likely to occur in the future. These predictions allow us to make sense of noisy or ambiguous inputs, make decisions despite uncertainty, and act efficiently and proactively, without having to wait for sensory feedback. Several influential theories posit that prediction may be a fundamental mechanism of brain function and behavior (e.g., Friston, 2010). Over the last decade, predictive frameworks have increasingly gained popularity to explain action, perception, cognition and even clinical disorders (e.g., Hudson et al., 2016; Corlett et al., 2019; Press et al., 2020; Kube and Rozenkrantz, 2021; and more for reviews). Within each of these fields, researchers have used a variety of techniques (from single cell recordings to psychophysics and human neuroimaging) and experimental paradigms (from repetition suppression to statistical learning) to try to elucidate predictive mechanisms. Although significant progress has been made in the study of predictive mechanisms within each of these domains, we have not yet begun to understand the parallels between them nor identify the key differences.

The goals of this Research Topic are to explore guiding principles, theoretical frameworks, and empirical research on predictive mechanisms across domains and levels of processing, with the ultimate goal of gaining a more comprehensive understanding of the limits, constraints, and generalizability of predictive mechanisms across fields. Empirical articles in this topic examined predictive processing using behavioral experiments and brain imaging across a number of domains including visual (Ueda et al.) and tactile processing (Beyvers et al.), proprioception (Fabre et al.), and auditory perception (Beach et al.). In addition, review articles applied predictive frameworks to higher-order domains such as social interaction (Neszmélyi et al.), reward, and self-concept (Mokady and Reggev).

Across the articles included in the Research Topic, some overarching themes emerge. Here, we discuss: (1) the creative applications and uses of predictive frameworks, (2) the ecological validity of predictive studies, and (3) the co-occurring stability and dynamism of predictions. Across each theme, we describe how the articles in this Research Topic contribute to furthering our understanding of the scope and limits of predictive frameworks to action, perception, cognition, and clinical disorders. Together, the articles here represent the unique breadth of predictive processing—ranging from action to disorders (as solicited by the title and call of the Research Topic).

## Predictive principles inform empirical study design, literature reviews, and hypothesis generation

The way researchers study, measure, and operationalize predictive processes varies greatly across and even within fields, with different insights to be gained depending on the methodology. For instance, across two different domains (auditory vs. tactile), Beach et al. and Beyvers et al. each take advantage of the principle that predictability modulates perception by suppressing predictable input in favor of mismatches, deviants, and surprising stimuli. Beyvers et al. examine detection thresholds on a tactile perception task to test the role of context and intensity in tactile suppression. Beach et al. combine magnetoencephalography (MEG) with an auditory repetition task, in which repetition of a syllable builds a neural representation of a “standard” against which future deviant inputs can be compared. These articles apply a similar core principle of predictive processing (i.e., suppression of predictable inputs) to vastly different domains, showcasing the universality of predictive frameworks and their influence on study design across fields.

Neszmélyi et al. and Mokady and Reggev apply predictive frameworks to respectively investigate discrepancies in existing literatures and propose new theories in higher-order cognitive domains. Neszmélyi et al. review studies that examine the content of social action representations, to develop a framework of how people plan and predict the consequences of their social interactions, while Mokady and Reggev apply predictive principles to put forth new hypotheses regarding self-concept as a product of stable, self-confirming priors built up over the lifetime.

This breadth in applications of predictive frameworks across domains emphasizes their power to influence research design and interpretation, highlights their concurrent generalizability and flexibility to account for a wide range of cognitive processes, and elucidates their capacity to generate testable predictions.

## Predictive processing in the wild: Showcasing the ecological validity of predictive frameworks

Fabre et al. and Ueda et al. each examine how restricted sensory feedback affects the brain and behavior in very practical ways. Predictive processing relies not only on top-down cues, but also on the integration of bottom-up sensory information. Fabre et al. examine standing and stepping behavior in a group of obese individuals in whom sensory feedback from the sole of the foot is reduced or restricted due to increased weight. The authors show that reducing body weight (i.e., “unweighting”) increases sensory evoked potentials in somatosensory cortex, equalizes the distribution of pressure on the foot, and decreases compensatory body-weight shifts to ultimately support predictive postural adjustments, balance, and stepping. Similarly, Ueda et al. compare performance on an in-lab task with real driving behavior to study the effect of predictions under restricted visual feedback. They find that real-life driving performance (i.e., steering smoothness) under visual restriction is related to participants' performance in in-lab tasks of restricted visual feedback, suggesting that participants utilize similar prediction-based models to overcome restricted visual feedback. These studies showcase the real-life applications of predictive frameworks.

## Predictions are both stable and dynamic

Predictive frameworks assume that when interpreting information or acting in the world, incoming information and existing predictions are weighted as a function of their reliability or precision. Estimates of reliability and precision can have different impacts on sensory processing and the malleability of the existing predictions. Several articles were unique in their exploration of the factors affecting predictions, with some suggesting that predictions were malleable and others suggesting they were quite stable. For instance, Beyvers et al., assessed tactile suppression using vibrating feedback to the finger on a reach task, and found that predictions were adaptive: the strength of tactile suppression was modulated not only by the predicted intensity of sensory outcomes, but also by their task-relevance. This is in line with the assumption that factors such as attention and utility can flexibly affect the weighting of predictions during sensory processing.

On the other hand, Mokady and Reggev suggest that predictions relating to the *self-concept* are uniquely stable, such that disconfirming evidence is interpreted in light of these self beliefs, rather than shifting the beliefs themselves. The authors propose that self-verification and self-enhancement interact with the self-concept to generate “stubborn” predictions. These predictions, in turn, influence the interpretation of information about the self, encouraging the maintenance of existing beliefs. This predictive “model of the self” provokes subjective reward responses, which further reinforce these beliefs.

Lastly, the stability of representations formed via predictive mechanisms are altered in disorders. Beach et al. use MEG to assess how neural representations of a “standard” against which deviants can be identified emerges and found neural differences in these representations in adults with and without dyslexia. While both groups were sensitive to stimulus repetition, increasing repetition history had a greater effect on the representation of a standard syllable in the neurotypical group than in the dyslexic group. The dyslexic group was less sensitive to the quantity of prior information in forming a prediction that might ultimately affect perception. This suggests that repetition contributes to the build-up of highly reliable expectations about forthcoming stimuli in neurotypicals, but less so in dyslexia.

## Conclusions and future directions

Together, the articles included in this Research Topic raise several testable hypotheses and directions for future research investigations. These include operationalization of terms used across domains (e.g., suppression), a deeper investigation of the proposed inverse relationship between precision and updating of predictions, applications of predictive frameworks in ecologically valid ways, and more direct tests of how these factors play out in different tasks within and across domains. This Research Topic showcases the strength of prediction frameworks in accounting for a wide range of cognitive processes, from higher order mechanisms of self and social cognition to fundamental processes of tactile perception during reaching, with direct applications to people's performance in everyday tasks.

## Author contributions

AD, PB, PC, and LR edited manuscripts in the Research Topic. AD and LR drafted the editorial. PB and PC provided feedback. All authors contributed to the article and approved the submitted version.

## Conflict of interest

The authors declare that the research was conducted in the absence of any commercial or financial relationships that could be construed as a potential conflict of interest.

## Publisher's note

All claims expressed in this article are solely those of the authors and do not necessarily represent those of their affiliated organizations, or those of the publisher, the editors and the reviewers. Any product that may be evaluated in this article, or claim that may be made by its manufacturer, is not guaranteed or endorsed by the publisher.
